# Distribution of Pb, Zn and Cd in stream and alluvial sediments in the area with past Zn smelting operations

**DOI:** 10.1038/s41598-021-96989-y

**Published:** 2021-09-02

**Authors:** Gorazd Žibret, Barbara Čeplak

**Affiliations:** Geological Survey of Slovenia, Dimičeva 14, 1000 Ljubljana, Slovenia

**Keywords:** Environmental sciences, Hydrology

## Abstract

The sources of Zn, Pb and Cd in alluvial and stream sediments have been studied in the area of historical Zn smelting tradition. 30 samples of stream sediments and samples from 4 alluvial sediment profiles were collected. Fractions 0.125–0.063 and < 0.063 mm were analysed by the means of ICP-MS prior 4-acid digestion. The highest levels of Zn, Cd and Pb were detected in the alluvial sediments in the closest vicinity to the abandoned slag and ore roasting residue waste dumps, reaching 96 and 4520 mg/kg, 522 and 26,800 mg/kg and 3.7 and 31 mg/kg for Pb, Zn and Cd in stream and alluvial sediments, respectively. The Voglajna River then transports contamination particles into the Savinja River, which afterwards flows into the Sava River. Consequently, the anomaly can even be detected in the Sava River, more than 30 km downstream. Higher levels of Pb, Zn and Cd have been found in fraction < 0.063 mm compared to 0.125–0.063 mm fraction. Impacts of historically contaminated soil erosion and in particular the wash-out of Zn-smelting waste from the improperly managed waste dump were recognised as the dominant sources of Zn, Cd and Pb in the stream and alluvial sediments.

## Introduction

Ore smelting activities represent one of the major sources of potentially toxic elements (PTEs) worldwide^1–5^. Although PTEs originate from the Earth's crust, human activities change their concentrations and distributions in the environment in a way that it is often difficult to distinguish between natural and anthropogenic contributions^6^. Dust emissions from smelters and wash-out from slag deposits in the past and present have led to the release of large quantities of PTEs into the environment^7^, which in many cases end up in the surrounding waters, soil and air^8,9^. The transport of PTEs by rivers is of particular concern because rivers can deposit these contaminants in their alluvial plains. Because of that, river sediments can act as a sink, and source of PTEs in the environment for the decades after the anthropogenic source is not active anymore. Additionally, river sediments keep records of past anthropogenic activities and they are often used as a tool to determine the total pollution in a certain area^10^.

Alluvial plains have been important agricultural areas globally, as well as the source of drinking water and dwelling place throughout human history. Nowadays alluvial sediments are contaminated due to past and present anthropogenic activities^11^, while the major contribution in many areas of the world can be attributed to mining and ore processing activities upstream^12,13^. The Danube River, which is a major waterway in Central Europe that developed vast alluvial plains and a large delta, is not an exception. Mining and ore processing activities in its catchment in the Alps, Carpathian, Dinarides throughout history had an important impact on the composition of its sediments.

The aim of this investigation is to assess the natural distribution of Pb, Zn and Cd in river sediment in the upper part of the river, to investigate the impact of anthropogenic activities on this natural distribution, in particular the erosion of contaminated soil and run-off from the poorly constructed pyrometallurgical waste deposit, and finally, to determine the influence area of anthropogenic impact and the rate of pollutant decrease in contaminated sediments in two fractions (< 0.063 mm and 0.063–0.125 mm) downstream the main source. These two fractions were selected because PTEs are commonly enriched in them and are best suited for studying river transport of contaminants and their deposition patterns. The authors also wanted to determine whether the transport and deposition mechanisms depends on the fraction size.

One of the major tributaries of the Danube is the Sava River, while the Savinja River, which is a tributary to the Sava River, is an important source of PTEs contaminated sediments in Sava^10^. The suspected anthropogenic source of PTE enriched particles in Savinja River could be connected to the historic legacy of Zn smelting (operational between 1870 and 1970) in Celje, the town on the banks of Savinja and Voglajna Rivers. Possible sources of Zn, Cd and Pb could be the erosion of historically contaminated soils, or wash out from improperly managed pyrometallurgical waste dump on the banks of Voglajna River (which is a tributary of Savinja), in the vicinity of the historic location of Zn smelting furnaces. Since the majority of Zn ore for the smelter was imported and there are no known larger ore deposits present in the area, the geogenic origin of this anomaly is highly unlikely. Considering the only dominant source of Pb, Zn and Cd is present in the Savinja catchment, this area is the suitable natural laboratory to achieve the aims of this study.

The knowledge gained in this study can be transferred to other historically contaminated and industrialised areas which are facing similar problems, and thus deepening the understanding of the behaviour of pollutants in river environments. It also highlights the importance of proper management of abandoned pyrometallurgical waste deposits and other historically contaminated sites and calls for proper environmental management of similar active sites globally.

## Materials and methods

### Study area

The beginning of the Zn-smelting operation in Celje dates back to 1873. Initial production facilities were upgraded in 1911 with the construction of a sulphuric acid production plant. The Celje zinc smelter processed mainly the sphalerite ore from Trepča (Serbia) and Titov Vales (Macedonia) mines. The local sources from nearby historical small-scale mines (Litija, Zavrh) were insignificant. The production capacity of the plant was significantly increased after World War II, but in 1970 the zinc smelting plant was shut down due to low prices of Zn and low supply of Zn ore from domestic (Yugoslavian) mines. Zn smelting was replaced by the titanium dioxide and other chemicals production, which have been in operation since then. The environmental legacy of historic Zn smelting in the area is the contaminated soil. The impacts of atmospheric dust emissions from Zn smelter were detected as far as 14–54 km in the attic dust and 9–14 km for Zn in the soil, depending on the direction from the plant^14^. Another legacy of historic Zn smelting is the pyrometallurgical waste deposit built-up (brownfield area) on the right bank of Voglajna River. 1–5 m thick wastes (slag, decayed fireproof material in ovens, tar, ash and other construction and demolition waste) were deposited directly on the alluvial sediments of the Voglajna River, without proper site preparation or measures to prevent PTEs mobilisation. The Pb and Zn contents in the upper anthropogenic soil layer reach 6 and 11% respectively, and Cd levels 344 mg/kg^15^. The material from this deposit is very likely washed out to the drainage network during high precipitation events.

Other important potential anthropogenic sources of Pb, Zn and Cd in the area are Železarna Štore ironworks, located 2 km southeast from the Zn smelting plant, and two active gypsum deposits in the Bukovžlak area, 2 km east from historic location of Zn smelting plant.

Historical data shows that the Voglajna and Savinja river sediments were enriched with Zn and Cr by a factor of more than 100, while the concentrations of Co, Pb and Cd were enriched by a factor of 25–50, 6 years after the end of Zn smelting in Celje^16^. In the year 2002, the sediments of Voglajna and Savinja showed the highest levels of Pb in the sediments near Štore, while the highest levels of Zn and Cd were detected in the sediments of the Savinja river about 5 km downstream from Celje in Tremerje^17^. A more recent study examining the sediments of the Sava River (Savinja is affluent of Sava) pointed to the increased concentrations of Pb, Zn and Cd in the stream sediments after the confluence of Savinja and Sava Rivers^10^. The most distinguished increase was detected for Cd levels in stream sediments (a fivefold increase compared to the natural geochemical background).

The river Savinja spring is located in the glacial valley of Kamnik-Savinja Alps in the Logarska Valley. The river flows through mountainous landscapes past the towns of Luče and Nazarje before reaching the Celje basin. After Celje, which is the largest town in its course, the river makes a sharp turn towards the south and cuts antecedent valley through the Sava Fold hills, where it flows into the river Sava at the village Zidani Most. The catchment area of the Savinja River basin covers 1864 km^2^ and in its 107 km of length, it drops for 750 m^18^. The river Savinja has several tributaries, one of the most important is the Voglajna River.

In the upper mountainous part, the average annual precipitation is 1700 mm, with increased snowfalls in the mountains, while in the middle and the lower part the average precipitation is around 1200 mm^19^. In 2020 the minimum, average and maximum discharge were 9.4, 33.5 and 392 m^3^/s respectively^20^. The Savinja River has an alpine rainfall-snowmelt regime. This regime has two maximums and two minimums. The main maximum occurs in early spring, while the second one is in autumn, usually in November. The main minimum is during the summer, more specifically in August, and the second minimum is in winter. The duration of the winter minimum is shorter^21,22^. The catchment area of the Savinja river is largely covered by forests and agricultural land. The Savinja river shows its torrential character along its entire length, with intense erosion sections in the upper mountainous part, well-developed alluvial plains in the Celje basin and again erosional character in antecedent flow through the Sava fold hills.

**Figure 1:**
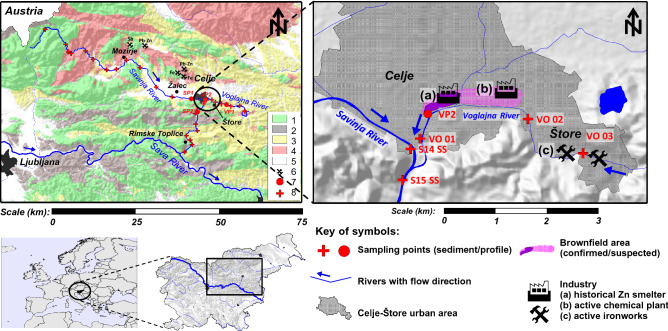
Map of the investigated area, its geological and morphological settings, location of samples and main potential anthropogenic sources of elements. 1—mainly Mesozoic carbonates (limestone, dolomite); 2—Paleozoic clastites; 3—Paleogene and Neogene sedimentary clastic rocks; 4—vulcanites and felsic igneous rocks of various ages; 5—Paleocene–Pleistocene alluvial sediments in basins; 6—small-scale metal mineralisation in the catchment area; 7—alluvial sediment sampling location (profiles), 8—stream sediments sampling locations. The maps were generated using Golden Software Surfer, ver. 21.1.158, https://www.goldensoftware.com/products/surfer.

The upper part of the Savinja river consists mainly of Triassic carbonate rocks (limestone and dolomite), while in the middle and lower section shales, tuffs, sandstones and claystones prevail^22,23^. Basins are filled with gravel, mainly of carbonate origin. The Voglajna river, with a length of 35 km in its upper reaches, flows in a more clastic environment—predominantly through Miocene sandstone, marl, claystone, with some intermediate sections of older tuff and felsic extrusive igneous rocks (rhyolite, trachyte)^23^ (Fig. 1). No larger metal mines are located in the Savinja catchment area, only small scale Sb–Pb–Zn-Fe deposits (Zavrh, Železno, Lepa Njiva, Puharje and Galicija) are present^24^. Their contribution to the elemental levels in stream and alluvial deposits is not expected to be detectable.

### Sampling and sample preparation

**Figure 2:**
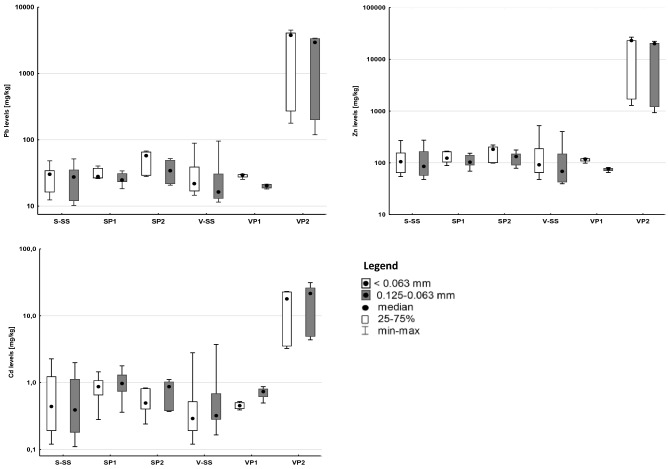
Range, quartiles and median of Pb, Zn and Cd content in Savinja and Voglajna stream sediments and alluvial sediment profiles upstream and downstream main source. *S-SS* Savinja stream sediments, *SP1/SP2* Savinja river alluvial sediments above/below contaminated site, *V-SS* Voglajna stream sediments, *VP1/VP2* Voglajna river alluvial sediments above/below contaminated site.

The sampling area can be divided into two parts: upstream of the main contaminated site of Celje, and the Celje area, and downstream of Celje. Sampling was conducted in July and August 2019. 23 stream sediments from the whole course of the Savinja River (S-01 to S-23), 7 samples from the entire course of the Voglajna River (V-01 to V-07), samples from 2 profiles through the recent alluvial sediment accumulation from the Savinja (SP1 and SP2) and Voglajna rivers (VP1 and VP2; 4 profiles in total) were collected (Table 1) up-and downstream from the main suspected causes of contamination (Figs. 1, 2).

**Table 1:** Number of samples/profiles above and below the contaminated site.

Stream sediments	No. of samples/profiles above the contaminated site	No. of samples/profiles below the contaminated site	Total number of samples	
Savinja	14	9	23	
Voglajna	4	3	7	

Alluvial sediment profiles	No. of samples/profiles above the contaminated site	Max depth of profiles (m)	No. of samples/profiles below the contaminated site	Max depth of profiles (m)
Savinja	1	0.9 (5 samples)	1	1.3 (7 samples)
Voglajna	1	0.7 (4 samples)	1	1.3 (7 samples)

The stream sediment samples represented fine sediments deposited in the active river channels after the last flood event. The material was collected with a spatula and a bucket on at least five different micro-locations on the inner convex river band. The samples were mixed and then stored in a plastic bag. The bucket and spatula were cleaned before the next use to prevent cross-contamination.

Alluvial sediment samples were collected by drilling through sediment accumulations on a convex river band between the river channel and flood protection dyke using a hand auger. The first sample was taken at a depth of 10 cm and the next one every 20 cm thereafter. The depths of the profiles ranged between 90 and 150 cm, depending on the location, and it ended in all cases when gravel river bed or anti-flood structures were reached. To minimise cross-contamination, the auger was cleaned after each collected sample, as well as the bottom of the hole was cleaned of fallen debris before a new sample was taken.

Stream and alluvial sediment samples were processed in the granulometric laboratories at the Geological Survey of Slovenia, where they were dried in the oven at 303 K. Plant remain, rocks, and other debris were removed. Particle aggregates were gently crushed in a ceramic mortar, taking care that skeletal particles remained intact. Dry screening was then carried out to obtain 2 pulps, each containing 5 g of fractions 0.125–0.063 mm and < 0.063 mm, which were afterwards used for elemental analysis.

Inductively coupled plasma mass spectrometry (ICP-MS) was performed after near-total 4-acid digestion at Bureau Veritas Mineral Commodities Laboratories, Vancouver, Canada, according to the international standards ISO 9001:2008 (analytical package MA250). Pulp containing material for analysis was stirred and 0.25 g split was heated in HNO_3_, HClO_4_ and HF to fuming and taken to dryness. The residue is dissolved in HCl. The presence of 60 elements was determined, however, only 3 (Pb, Zn and Cd) are presented in this paper, because only three elements show the largest variations between their natural and anthropogenic distributions.

The precision of the elemental analysis was controlled with 15 duplicates for Pb and Cd, and with 16 duplicates for Zn, while the accuracy was determined on the basis of 17 analysis of 7 standard materials (laboratory internal standards OREAS25A-4A, OREAS45E and OREAS45H and the certified reference materials by European Joint Research Centre BCR-723 road dust, BCR-176R fly ash, BCR-320R channel sediment and BCR-142R soil). The calculated quality control parameter for precision is the relative percentage difference (RPD; Eq. 1).

1 $$\mathrm{RPD }=\frac{100}{10} {\sum }_{n=1}^{10} \frac{2|{CM}_{n}- {CR}_{n}|}{{CM}_{n}+ {CR}_{n}}$$

CM—measured concentrations, CR—concentrations in the duplicate.

The average percent recovery rate (%R) was a measure for the accuracy of chemical analyses (Eq. 2).

2 $$\mathrm{\%R}=\frac{100}{5} \cdot {\sum }_{n=1}^{5} (1+\frac{{CM}_{n}-{CS}_{S}}{{CS}_{s}})$$

CM—measured concentrations, CS—known elemental level in the standard.

The bias (B) of the analyses was measured by the analysis of 6 blank samples (triple distilled water) and is presented as the average concentration of elements in blanks.

### Data analysis

Basic statistical parameters were calculated for 3 elements in both of the analysed fractions (0.125–0.063 mm and < 0.063 mm) for stream and alluvial sediments of Savinja and Voglajna Rivers. In the case double analysis were made for precision control, the true value was assumed to be the average value of both analyses.

Several different enrichment ratio (ER) parameters were used to compare measured values with the reference ones. The first one was the ER_EU_ representing enrichment of the Savinja and Voglajna River stream and alluvial sediments with the corresponding European median values^25^ (Eq. 3).

3 $${\text{ER}}_{{{\text{EU}}}} = {\text{Md}}_{{\text{x}}} /{\text{Md}}_{{{\text{EU}}}}$$

ER_EU_—enrichment ratio of median elemental levels according to the European stream sediment median elemental levels, Md_x_—median of the corresponding material (stream or alluvial sediment of Savinja or Voglajna) and Md_EU_—the corresponding European stream sediment median^25^.

The ER_frac_ was calculated by comparing median values for both analysed fractions (Eq. 4).

4 $${\text{ER}}_{{{\text{frac}}}} = {\text{Md}}_{{0.{125} - 0.0{63}}} /{\text{Md}}_{{ < 0.0{63}}}$$

ER_frac_—enrichment ratio of median elemental levels in the 0.125–0.063 mm fraction compared to the median elemental levels in the < 0.063 mm fraction.

Md_0.125–0.063_—medial elemental level in the 0.125—0.063 mm fraction.

Md_<0.063_—median elemental level in the < 0.063 mm fraction.

The third ER_source_ represented elemental enrichment downstream of Celje, compared to the estimated natural background levels in this study (Eq. 5).

5 $${\text{ER}}_{{{\text{source}}}} = {\text{Md}}_{{{\text{downstream}}}} /{\text{C}}_{{{\text{background}}}}$$

ER_source_—enrichment ratio of elemental levels downstream of the main source of the pollution compared to the corresponding geochemical background values, Md_downstream_—median elemental level downstream of the source of the pollution and C_background_—obtained geochemical background value.

Elemental levels measured in the sediments have been plotted according to their relative positions in the river course, and elemental levels in sediments according to their depths. Background values for stream sediments for the study area have not been established yet. For the purpose of indices calculations, background values were estimated from the elemental analysis of stream sediments in the uppermost of Savinja river from this study.

### Pollution indexes

To assess the state of pollution, the pollution load index (PLI) and the Nemerov index (I_N_) were calculated. These indexes are widely used to assess the global pollution level^26^. The PLI proposed by Tomlinson et al.^27^ represents the geometric mean of the values of the Single Pollution Index (PI) for several elements, which is used to assess the degree of pollution of individual pollutants in sediments. The PI is the ratio between the specific elemental level in the sediment and the corresponding geochemical background value (Eq. 6). If the value of PLI (Eq. 7) is smaller than 1, the sediment is considered as uncontaminated, while values 1 < PLI < 2 point out to unpolluted to moderately, 2 < PLI < 3 to moderately to highly, 4 < PLI < 5 to highly and PLI > 5 to very highly polluted material^28^. The PI and PLI were calculated by the following equations:

6 $$PI={C}_{i}/{C}_{i,background}$$

7 $$PLI=\sqrt[n]{({{PI}}_{1} x {{PI}}_{2} x\dots x {{PI}}_{n})}$$

PI—single pollution index, PLI—pollution load index, C_i_—elemental level, C_i,background_—background elemental level and n—number of elements.

The geoaccumulation index given by Müller^29^ is calculated by Eq. 8 and was used for the calculation of Nemerov index (Eq. 9), which is more suitable for the cases where contamination of larger areas are assessed.

8 $${I}_{geo}={log}_{2}(\frac{{C}_{i}}{1.5{C}_{i,background}})$$

I_geo_—geoaccumulation index, C_i_—elemental level and C_i,background_—background elemental level

9 $${I}_{N}=\sqrt{\frac{{I}_{geomax}^{2}+{I}_{geoavg}^{2}}{2}}$$

I_N_—Nemerov index, I_geomax_—is the maximum I_geo_ and I_geoavg_- is the average value of I_geo_.

According to Förstner et al.^30^ the classification of I_N_ is as follows: 0 < I_N_ ≤ 0.5, uncontaminated; 0.5 < I_N_ ≤ 1, uncontaminated to moderately contaminated; 1 < I_N_ ≤ 2, moderately contaminated; 2 < I_N_ ≤ 3, moderately to heavily contaminated; 3 < I_N_ ≤ 4 heavily contaminated; 4 < I_N_ ≤ 5, heavily to extremely contaminated; and I_N_ > 5, extremely contaminated.

## Results

Table 2 and Fig. 2 show the descriptive statistical parameters (minimum, maximum and median values) of elemental levels for all samples in two size fractions (0.125–0.063 and < 0.063 mm), the European background levels for stream sediments^25^, estimated background levels for this survey and the quality control parameters (RPD, %R and B). Analytical values are presented in Table S1. The median values of Pb, Zn and Cd of stream sediments are comparable to the corresponding European stream sediment median levels. Alluvial sediments are generally enriched with Pb, Zn and Cd compared to stream sediments. This is especially valid for the Voglajna River alluvial sediments where the highest levels of Pb, Zn and Cd in this study have been detected—Zn levels reach % range, surpassing even corresponding maximum level of European alluvial sediments.

**Table 2:** Descriptive statistical parameters of Pb, Zn and Cd levels (mg/kg) in stream and alluvial sediments collected in Savinja and Voglajna Rivers in two analysed grain size fractions.

	*R *(%*)*	*ARPD *(%)	*B *(%)	Size of fraction (mm)	S-SS (mg/kg)	V-SS (mg/kg)	SP (mg/kg)	VP (mg/kg)	*E-SS (mg/kg)*	*BGV (mg/kg)*
Pb	98	8	0	< 0.063	12–48 (30)	15–89 (22)	26–68 (35)	25–4500 (270)	N.A	15
				0.125–0.063	10–52 (27)	12–96 (16)	18–52 (28)	18–3400 (200)	< 1.0–5760 (20.5)	12
Zn	99	7	0.17	< 0.063	54–270 (105)	48–520 (92)	88–220 (140)	98–27,000 (1700)	N.A	63
				0.125–0.063	48–270 (85)	39–400 (68)	69–180 (120)	65–22,000 (1200)	4.0–13,900 (71.0)	57
Cd	63	14	0	< 0.063	0.11–2.0 (0.39)	0.17–3.7 (0.32)	0.36–1.8 (0.91)	0.50–31 (4.9)	N.A	0.18
				0.125–0.063	0.12–2.3 (0.44)	0.12–2.8 (0.29)	0.24–1.5 (0.65)	0.39–23 (3.5)	< 0.02–43.1 (0.28)	0.17

**Figure 3:**
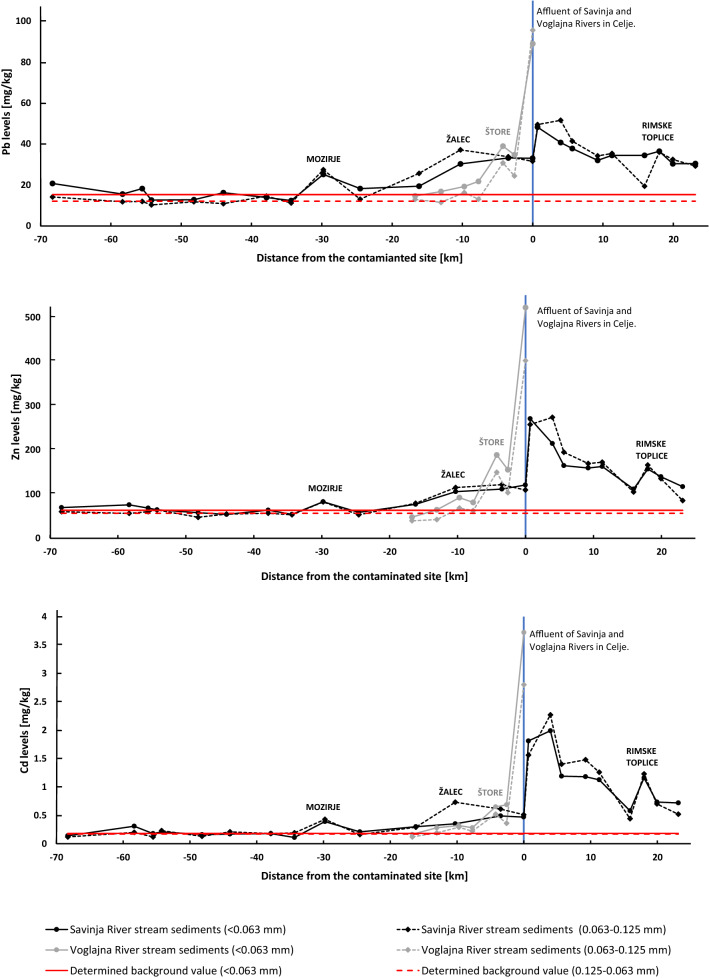
Pb, Zn and Cd levels in stream sediments relative to the position of the Savinja and Vogjalna affluent. The pyrometallurgical waste deposit is located on the right bank of the Voglajna river, approx. 1 km upstream Savinja and Voglajna rivers. X-axis presents distance according to the water flow*.*

The presentation format is: minimum value–maximum value (median value), *R* average percent recovery, *ARPD* average relative percent difference between double analysis, *B* bias, *N.A.* not analysed, *S* Savinja, *V* Voglajna, *E* data from the European stream sediment survey^25^, *SS* stream sediments, *P* alluvial sediments, *BGV* estimated background value from the data in this study (details are shown in Fig. 3).

The values of ER_EU_ parameter (Table 3) show that Pb, Zn and Cd levels in the stream sediments of Savinja and Voglajna Rivers and values of alluvial sediment of Savinja River are comparable to the corresponding median values of sediments in Europe. However, median Pb, Zn and Cd levels in Voglajna river alluvial sediments are 9.8, 17 and 13-fold higher than the corresponding European values. ER_frac_ values (Table 3) show Pb, Zn and Cd enrichments in smaller fraction compared to coarser ones, while the values of ER_source_ show significant enrichments downstream Celje, compared to corresponding upstream values. ER_source_ value for Cd reaches 7.1 in the fraction 0.125–0.063 mm in stream sediments and 15 in the < 0.063 mm fraction in alluvial sediments.

**Table 3:** Enrichment ratios for stream and alluvial sediments.

Stream sediments							Alluvial sediments					
Element	*ER*_*EU*_		*ER*_*frac*_		*ER*_*source*_		*ER*_*EU*_		*ER*_*frac*_		*ER*_*source*_	
	*S*	*V*	*S*	*V*	< 0.063 mm	0.125–0.063 mm	*S*	*V*	*S*	*V*	< 0.063 mm	0.125–0.063 mm
Pb	1.3	0.78	0.9	0.7	2.4	2.9	1.4	9.8	0.8	0.7	8.0	7.1
Zn	1.2	0.96	0.8	0.7	2.6	2.9	1.7	17	0.9	0.7	12	9.7
Cd	1.6	1.0	1.1	0.9	6.1	7.1	2.3	13	0.7	0.7	15	12

*S* Savinja River, *V* Voglajna River, *ER*_*EU*_ enrichment ratio of median elemental levels according to the European stream sediment median elemental levels, *ER*_*frac*_ enrichment ratio of median elemental levels in 0.125–0.063 mm fraction compared to the median elemental levels in < 0.063 mm fraction, *ER*_*source*_ enrichment ratio of elemental levels downstream of the main source of the pollution compared to the estimated corresponding geochemical background values.

Pb, Zn and Cd levels in Savinja stream sediments in the upper part (until the town of Mozirje) are more or less constant, and corresponding average values can be regarded as the natural background level (Fig. 3). Approaching the town Žalec Pb, Zn and Cd levels start to steadily increase, until the affluent of Voglajna River, where the sharp rise of all three elemental levels has been observed. The highest Pb, Zn and Cd levels in the Savinja River stream sediments were measured in the first two samples, located downstream of the affluent with Voglajna River, reaching 48 and 52, 270 and 270, and 2.0 and 2.3 mg/kg for < 0.063 and 0.125–0.063 mm fractions, respectively (Table 2). Pb, Zn and Cd levels slowly decrease downstream until the affluent with the Sava River. However, background levels are not reached anymore.

A similar pattern is observed in the case of Voglajna River stream sediments (Fig. 3). In the upper part Pb, Zn and Cd levels are comparable to the estimated natural background. A steady rise is observed as the river approach the populated area of Celje. The first increase is observed nearby Štore ironworks, and the second larger increase is observed downstream of the pyrometallurgical waste dump. These are the highest measured Pb, Zn and Cd levels of stream sediments in this study, reaching 89 and 96, 520 and 400, and 3.7 and 2.8 mg/kg for the < 0.063 and 0.125–0.063 mm fractions, respectively (Table 2).

**Figure 4:**
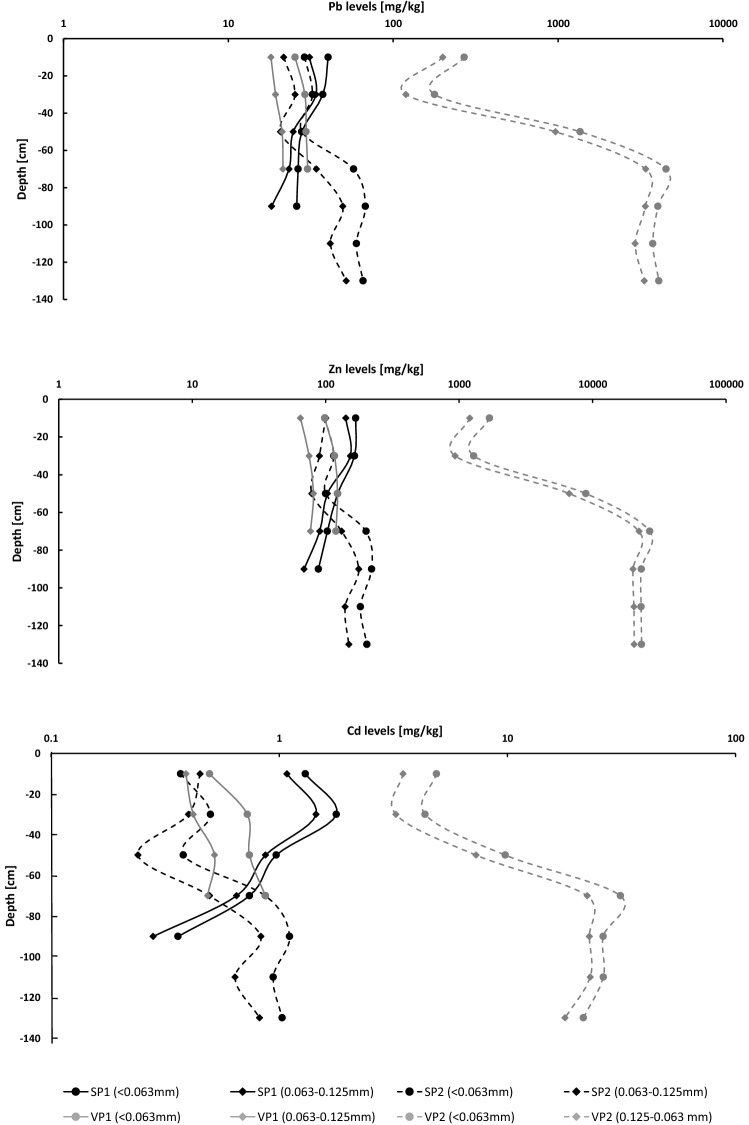
Pb, Zn and Cd levels in alluvial sediment profiles. *SP1/SP2* alluvial sediment profile from the Savinja river floodplain above/below Celje, *VP1/VP2* alluvial sediment profile from the Voglajna river floodplain above/below Celje.

The samples from alluvial sediment profiles were taken upstream (SP1 and VP1) and downstream (SP2 and VP2) from the pyrometallurgical waste dump. The expected Pb, Zn and Cd levels in profiles SP2 and VP2 should therefore be higher than the corresponding levels in profiles SP1 and VP1. The results show that comparable levels of Pb, Zn and Cd are found in profiles SP1, SP2 and VP1, varying between 18 and 68, 65 and 221 and 0.24 and 1.8 mg/kg for both fractions respectively, while corresponding levels in profile VP2, which is located in the immediate vicinity of the waste deposit are for a magnitude higher (Fig. 4). Pb, Zn and Cd levels in the coarser fraction in the upper part of the profile are around 220, 1500 and 4.6 mg/kg, while in the lower part extreme values were detected (4100, 24,000 and 26 mg/kg, respectively). Pb, Zn and Cd levels in < 0.063 mm fraction in all four profiles are between 20 and 50% higher than corresponding levels in 0.063–0.125 mm fraction (Fig. 4). These results pointed out a dominant anthropogenic source of Pb, Zn and Cd in the stream and alluvial sediments in the study area.

The contamination of stream and alluvial sediments downstream of the pyrometallurgical waste dump was assessed using PLI and I_N_ indexes (Table 4). According to the PLI value, both stream and alluvial sediments of both rivers in their lower part could be regarded as contaminated. The highest values of I_N_ index were detected for Cd, showing that stream sediments can be considered as moderately to heavily contaminated, alluvial sediments of Savinja River as moderately contaminated, while alluvial sediments of Voglajna River as extremely contaminated (Table 4).

**Table 4:** Calculated PLI and I_N_ indexes for stream and alluvial sediments downstream the dominant source of contamination for both fractions.

	N	PLI_Min_	PLI_Md_	PLI_Max_	I_N_ (Pb)	I_N_ (Zn)	I_N_ (Cd)
**Stream sediments**							
Savinja (mm)							
< 0.063	9	2.4	3.3	5.2	0.91	1.2	2.5
0.125–0.063	9	1.7	3.6	5.7	0.96	1.2	2.6
Voglajna (mm)							
< 0.063	3	2.8	3.0	10.0	1.6	2.0	3.1
0.125–0.063	3	1.7	2.4	8.6	1.6	1.6	2.6
**Alluvial sediments**							
Savinja (mm)							
< 0.063	7	1.8	3.9	4.6	1.3	0.99	1.7
0.125–0.063	7	1.3	2.4	3.5	0.94	0.68	1.3
Voglajna (mm)							
< 0.063	7	18	230	280	6.9	7.4	6.3
0.125–0.063	7	13	190	220	6.5	7.1	5.9

*N* number of samples considered, *Min* minimum value, *Md* median, *Max* maximum value.

## Discussion

### Pb, Zn and Cd levels upstream waste deposit

The spatial distribution of elemental levels of Pb, Zn and Cd reveal that the area can be divided into two parts: upstream and downstream Celja, and in particular upstream/downstream pyrometallurgical waste dump, where sharp rise of Pb, Zn and Cd levels in stream and alluvial sediments have been found. The I_N_ index indicates moderate to heavy contamination of Savinja stream and alluvial sediments, while Voglajna alluvial sediments next to the pyrometallurgical waste deposit can be classified as extremely contaminated.

In order to determine the significance of detected contamination, maximum Pb, Zn and Cd levels from this study were compared to values, obtained by other studies around the globe (Table 5). The comparison reveals that maximum Pb, Zn and Cd levels in stream and alluvial sediments of the Savinja River are generally lower than from similar sites around the world. However, the maximum Pb, Zn and Cd levels in alluvial sediments of the Voglajna River are comparable to those found at the most contaminated sites globally.

**Table 5:** Maximum elemental levels in stream and alluvial sediment samples from the present and similar studies globally.

River, country	Fraction size (µm)	Chemical analysis	The main anthropogenic source of Pb, Zn and Cd	Max (mg/kg)			References
				*Pb*	*Zn*	*Cd*	
**Stream sediments**							
Savinja, Slovenia	< 63	TD, ICP-MS	Abandoned Zn-smelter, Celje	48	270	2	This study
Voglajna, Slovenia	< 63	TD, ICP-MS	Abandoned Zn-smelter, Celje	89	520	3.7	This study
Sava, Slovenia	< 63	AR, ICP-MS	Abandoned smelter Litija, iron mines Savska Jame, abandoned Zn-smelter Celje	58.4	139	0.8	^10^
Vardar, Macedonia	< 125	ICP-MS, ICP-AES, TD	Abandoned Pb–Zn smelter Vales, active Pb–Zn mines Zletovo and Toranica	34	83	0.43	^4^
Streams in Guizhou province, China	< 125	sequential extraction analysis, mixed acid digestion (HNO_3_, HF), AAS	Abandoned Zn–Pb smelter, Hezhang Country	21,850	30,425	97	^5^
Rambla de La Morera, Spain	< 180	TD, ICP-MS	Abandoned Pb–(Ag)–Zn mining and metalurgical activities, Mazarrón	10,100	3400	10.5	^3^
Xiangjiang river, China	< 150	TD, ICP-MS	Abandoned Pb–Zn mining and smelting activities, Hunan province	672	1010	31.2	^1^
Hurtado river, Chile	< 64	AR, AAS	As–Cu–Zn mineral deposits, Coquimbo region	–	6580	31.4	^2^
Litavka, Czech Republic	< 63	sequential extraction analysis, mixed acid digestion (HF, HClO_4_, HCl), FAAS	Abandoned Pb–Ag–Zn mining, smelting activities, Příbram	9800	26,039	316	^31^
Lăpuş, Romania	< 63	AR, ICP-OES	Abandoned mining sites, Băiuţ plants	879	4251	49.4	^32^
**Alluvial sediments**							
Savinja, Slovenia	< 63	TD, ICP-MS	Abandoned Zn-smelter, Celje	68	220	1.8	This study
Voglajna, Slovenia	< 63	TD, ICP-MS	Abandoned Zn-smelter, Celje	4500	27,000	31	This study
Drava, Slovenia	< 200	TD, ICP-MS	Abandoned Zn–Pb mining and smelting (Mežica, Cave del Predil, Bleiberg-Kreuth)	1200	3300	17	^33^
Wurm River, Germany	< 63	XRF	Abandoned Pb–Zn-smelting and mining activity, Stolberg	1052	2575	–	^34^
Litavka River, Czech Republic	< 200	Sequentual extraction analysis, mixed acid digestion (HF, HClO_4_), FAAS	Abandoned Pb–Ag–Zn mining, smelting activities, Příbram	4705	8728	67.5	^35^
Trent catchment, UK	< 200	TD, ICP-MS	Abandoned Pb–Zn mining and smelting industry, Southern Pennine Orefield	1300	2000	22	^36^
Lăpuş, Romania	< 63	AR, ICP-OES	Abandoned mining sites Băiuţ plants	5050	1050	12	^32^

*AAS* atomic absorption spectrometry, *FAAS* flame absorption atomic spectrometry, *ICP-MS* inductively coupled plasma mass spectrometry, *ICP-AES* inductively coupled plasma atomic emission spectrometry, *ICP-OES* inductively coupled plasma optical emission spectrometry, *AR* aqua regia digestion, *TD* total acid digestion.

Pb, Zn and Cd levels in the upper parts of the Savinja and Voglajna Rivers very likely resemble natural background values. The only exception is a slight increase in Pb, Zn and Cd levels in stream sediments at the town Mozirje. Metals, wood and plastic processing industry and agriculture are present there, as well as the emissions from associated traffic, all of them potentially contributing to the metal intake into the drainage network^37,38^. Pb, Zn and Cd levels after the town Mozirje drop to the background levels, so this increase was not found to be of great significance for this study.

Approaching Celje, past the town Šempeter, Pb, Zn and Cd levels begin continously increasing, especially for Pb. This is the area where the river reach basin with more intensive agriculture and urbanisation. River also flows parallel with the main highway connection A1/E57. Discharge from water treatment plants from the A1 motorway, as well as the wash-out of road dust, enriched with Pb, Zn and Cd from other paved areas^39,40^ can also be one of the reasons for the increase of these elements in that area^10,41,42^. Farming (especially hop farming) in the lower Savinja basin area can also contribute to the increased values of Pb, Zn and Cd, since fertilizers and phytopharmaceuticals could be enriched with these metals^38,43^.

As the Savinja River approaches the town Celje, the wash-out of the contaminated soil due to atmospheric dust deposition emitted from historical Zn smelter in Celje can be added to the list of potential sources^15,44,45^. The study of Žibret and Šajn^14^ in particular discovered that the effect of this smelter in the soil can be detected up to 14 km away. Curiously, Pb, Zn, and Cd levels in alluvial sediments in the Voglajna River follow the same pattern as the Savinja River—it shows a steady increase of Pb, Zn, and Cd levels as the river approaches Celje. The impact of the Štore steel mill also cannot be neglected in this case, as evident from the Fig. 3.

The study of Zhao et al.^9^ reported that the wash-out of historically contaminated soil can have a significant impact on the composition of stream sediments and that the highest levels of Zn, Pb and Cd in stream sediments of Baiyin district were detected around the non-ferrous mine and smelter. Huang et al.^46^ confirmed that the sediments from Huixian wetlands are 11% more polluted than nearby soils, the reason for this is the soil wash-out from contaminated site, transportation and sedimentation in wetlands downstream. The study of Balabanova et al.^47^ reported that particles originating from contaminated soil that enter the Zletovska River due to the nearby Pb–Zn mine, can also be detected several tens of km downstream of the Bregalnica River.

### Metal levels downstream pyrometallurgical waste deposit

**Figure 5:**
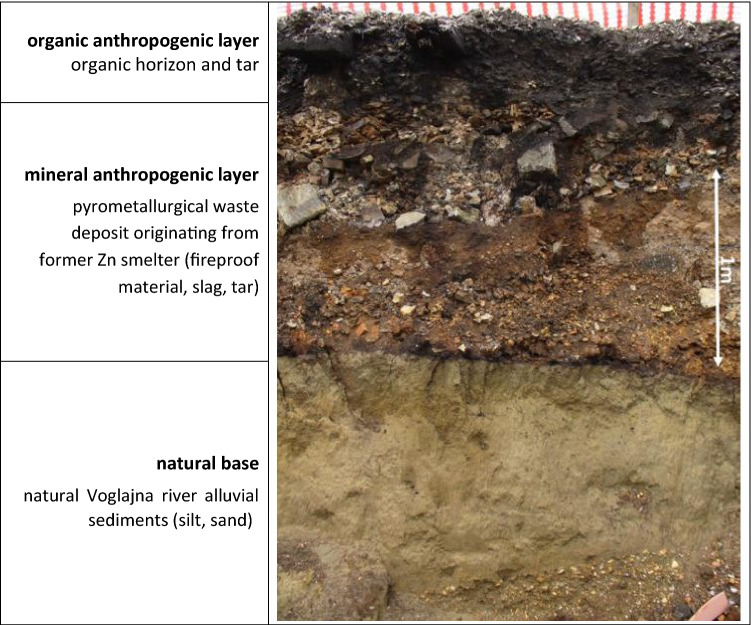
The characteristic profile through pyrometallurgical waste deposit (photo G. Žibret). The thickness of organic and mineral anthropogenic layers and their order of appearance in the profile can vary through the deposit.

The spatial distribution of Pb, Zn and Cd in stream and alluvial sediments clearly reveal that the pivotal source of Pb, Zn and Cd in the alluvial and stream sediments in the study area is the wash-out of contaminated particles from improperly constructed and managed pyrometallurgical waste dump. During the operation of the Zn smelter between 1870 and 1970, a large amount of waste was gradually piled up in the surroundings of the former smelter. As a result, slag, ashes, dust, construction waste (fireproof materials i.e. bricks from ovens and similar waste) and tar from the adjacent coking plant can be found throughout the area around Zn smelter^48^. Waste was placed directly on the Voglajna river alluvial sediments (Fig. 5), and it is still not protected from erosion in the case of heavy rain events, while the drainage is also not properly managed. Therefore, it is not a surprise that the highest values of Pb, Zn and Cd in stream and alluvial sediments in this study were found on the sampling point, located in the close vicinity donwnstream of this dump. Zn levels reached 27,000, Pb levels 4500 and Cd levels 32 mg/kg. Such conclusion can be further supported by the individual particle observation under SEM/EDS by comparing particles, carriers of PTEs in stream and alluvial sediments with corresponding particles located in the waste deposit by the future studies.

Just a few km downstream of this waste dump, the Voglajna River flows into a larger Savinja River. The impact of intake of contaminated sediments by the Voglajna River is clearly visible also in Savinja case, because the highest recorded Pb, Zn and Cd levels in Savinja River stream sediments are detected just after the confluence between Savinja and Voglajna rivers. Concentrations of investigated elements gradually decrease downstream, but they did not reach values similar as were prior to Celje before the confluence with Sava river. Interestingly, the sharp rise of Pb, Zn and Cd levels in stream sediments of regionally important Sava River can be further detected downstream the confluence with Savinja^10^.

It was discovered by many studies, that improperly managed mining and pyrometallurgical waste dumps can have a serious adverse impact on the composition of stream and alluvial sediments. During the Aznalcóllar dam collapse (Spain) in 1998 around 5 million cubic meters of acid waste from the processing of pyrite ore were released into the environment due to improper tailings management. In addition to the pollution of river sediments, alluvial plain and aquatic wildlife, the waste also entered into Doñana national park^49^. Similar tailing dam collapse happened in 2003 in North Macedonia at Sasa mines, where tailings enriched with Pb, Zn and Cd subsequently flew through the Kamenica River straight into Lake Klaimanci^50^. Similar accidents happened in Mount Polley gold and copper mine in British Columbia–Canada in 2014^51^, in an iron mine in Southeastern Brazil in 2015^52^ and elsewhere.

The fluvial dispersion of pollutants from historical Zn smelting site (at least 30 km away from the source) can therefore be detected over much longer distances compared to the impacts of atmospheric dispersion, which are estimated between 9 and 14 km for Zn and 6 and 32 km for Cd^14^. This result is also in agreement with other studies. Foucher et al.^53^ discovered, that the impacts of abandoned Hg mine can be detected even 500 km away from the mine, where the main dispersion mechanism was water transport (river and marine). A similar situation was also found in the study of Periáñez^54^, where coastal waters transported dissolved heavy metals from the Odiel-Tinto rivers as far as 200 km from the source of contamination.

### Pb, Zn and Cd levels from alluvial sediment profiles

It was expected that profiles above the sources of contamination (SP1 and VP1) should have lower Pb, Zn and Cd levels than profiles below Celje (SP2 and VP2). Pb, Zn and Cd levels at the upper part of the profile SP1 were slightly increased, which could be attributed to the use of Cd-enriched fertilisers^38^ and wash-out of contaminated soil. It was reported that a single flood event can produce a layer of fresh alluvial deposit as thick as 10.7 mm, depending on the frequency of flooding, erosion rate, etc^55^. The distribution of Pb, Zn and Cd levels in the profile SP2 shows that lower levels were detected in upper 50 cm, and sharp rise of pollutant levels in depthts greater than 50 cm. This result can be explained by the sedimentation of around 50 cm of fresh and less uncontaminated alluvial sediment after the cease of Zn production in Celje in 1970 during the floods in 1990, 1998, 2007, 2010, 2012 and 2017^56^. However, Pb, Zn and Cd levels in the lower parts of SP2 profile (below 50 cm) are around two-fold higher than the corresponding levels in stream sediments below Celje, and this material could have been deposited during the Zn-smelting operations between 1870 and 1970.

The vertical distribution of Pb, Zn and Cd in the profile VP2 is similar to that of the SP2 profile, with the difference that they are of a magnitude higher. The material from the upper 50 cm of VP2 profile, which has very high Pb, Zn and Cd levels, was probably deposited after 1970, while the material from the lower part of the profile reaches extreme values and very likely represent the material which was eroded from waste deposit during smelter operations.

The analysis of the construction trenches dug through this waste deposit showed that the area is covered with 0.5–4.7 m thick debris of construction waste, slag, bricks, slags and boilers^57,58^ (Fig. 5), which are placed directly on the permeable Voglajna river alluvial sediments. The past analysis also shows extreme Pb, Zn and Cd levels in these materials, even those above 1% for Zn^15^, and this data could further support the result of current study, that the major source of Pb, Zn and Cd levels in fluvial sediments of the Voglajna and Savinja rivers is actually the wash-out of the material from waste dump.

Pb, Zn and Cd levels in finer fraction < 0.063 mm of alluvial sediments are on average around 30% higher than in coarser fraction 0.125–0.063 mm, while for stream sediments this enrichment is less significant, around 10–15%. This was expected, because the levels of heavy metals in alluvial sediments generally increase with the decreasing particle size^59^.

## Conclusions

The focus of the study was to determine the sources of Zn, Cd and Pb in the fluvial system of the Savinja River. Samples of stream sediments were collected from the Savinja and Voglajna rivers source to the river mouths, while the samples of alluvial sediments were collected in alluvial profiles up-and downstream of the main suspected anthropogenic source, the abandoned pyrometallurgical waste deposit in Celje town. The majority of the processed ore in abandoned Zn smelter was imported, and no known larger Pb or Zn mines existed in the area. It was discovered that median values of stream sediments in this study are comparable to those of the representatives of European rivers, while they are generally lower than the values measured next to abandoned mines and smelters worldwide. In the upper parts of the Savinja and Voglajna rivers, Pb, Zn and Cd levels are constant and are clearly pointing to the natural background levels. A steady rise in Pb, Zn and Cd levels is observed as the rivers flow through densely populated areas with intensive agriculture. A sharp rise of Pb, Zn and Cd levels in stream sediments of the Savinja River after the confluence of the Voglajna river has been also observed. According to the geochemical indexes (PLI and I_N_) these sediments can be regarded as contaminated. Spatial distribution of measured levels reveals that the material wash-out from the improperly managed pyrometallurgical waste dump can be recognised as the main source of metal pollution of river sediments in this area because extreme values of Pb, Zn and Cd levels in the alluvial sediments of Voglajna River were measured next to this brownfield area. The wash-out from this dump is clearly affecting also the composition of stream sediments in Savinja River downstream the confluence with the Voglajna River. Although the Zn smelter in Celje has been shut down for half a century, the impacts of improper waste management are still present. The results of this study point out the need for proper management of abandoned and active pyrometallurgical and mine waste deposits, particularly for areas that are prone to erosion, to protect both human health and the health of other living organisms downstream.

## Supplementary Information

Supplementary Information 1.

## References

[1] Chai L (2017). Heavy metals and metalloids in the surface sediments of the Xiangjiang River, Hunan, China: Distribution, contamination, and ecological risk assessment. Environ. Sci. Pollut. Res..

[2] Oyarzun R, Lillo J, Oyarzún J, Higueras P, Maturana H (2006). Strong metal anomalies in stream sediments from semiarid watersheds in northern Chile: When geological and structural analyses contribute to understanding environmental disturbances. Int. Geol. Rev..

[3] Oyarzun R (2011). The Mazarrón Pb-(Ag)-Zn mining district (SE Spain) as a source of heavy metal contamination in a semiarid realm: Geochemical data from mine wastes, soils, and stream sediments. J. Geochem. Explor..

[4] Popov SI, Stafilov T, Šajn R, Tănăselia C (2016). Distribution of trace elements in sediment and soil from river Vardar Basin, Macedonia/Greece. Sci. Health Part A Toxic/Hazardous Subst. Environ. Eng..

[5] Yang Y (2010). Lead, Zn, and Cd in slags, stream sediments, and soils in an abandoned Zn smelting region, southwest of China, and Pb and S isotopes as source tracers. J. Soils Sedim..

[6] Belhadj H, Aubert D, Dali Youcef N (2017). Geochemistry of major and trace elements in sediments of Ghazaouet Bay (western Algeria): An assessment of metal pollution. Comptes Rendus Geosci..

[7] Wang J (2020). Geochemical transfer of cadmium in river sediments near a lead-zinc smelter. Ecotoxicol. Environ. Saf..

[8] Gutiérrez M, Mickus K, Camacho LM (2016). Abandoned Pb Zn mining wastes and their mobility as proxy to toxicity: A review. Sci. Total Environ..

[9] Zhao X (2020). A comprehensive investigation of hazardous elements contamination in mining and smelting-impacted soils and sediments. Ecotoxicol. Environ. Saf..

[10] Žibret G, Gosar M (2017). Multi-elemental composition of the Sava River sediments (Slovenia, EU). Environ. Earth Sci..

[11] Zimmermann T (2020). Zinc isotopic variation of water and surface sediments from the German Elbe River. Sci. Total Environ..

[12] Pavlowsky RT, Lecce SA, Owen MR, Martin DJ (2017). Legacy sediment, lead, and zinc storage in channel and floodplain deposits of the Big River, Old Lead Belt Mining District, Missouri, USA. Geomorphology.

[13] Pavlović P (2019). Evaluation of potentially toxic element contamination in the riparian zone of the River Sava. CATENA.

[14] Žibret G, Šajn R (2008). Modelling of atmospheric dispersion of heavy metals in the Celje area, Slovenia. J. Geochem. Explor..

[15] Voglar GE, Leštan D (2010). Solidification/stabilisation of metals contaminated industrial soil from former Zn smelter in Celje, Slovenia, using cement as a hydraulic binder. J. Hazard. Mater..

[16] Štern J, Förstner U (1976). Heavy metals distribution in the sediment of the Sava Basin in Slovenia. Geologija.

[17] Frančišković-Bilinski S, Bilinski H, Tibljaš D, Hanžel D, Mertelj A (2002). Characterization of sediments from Voglajna and Savinja rivers—preliminary results. Geologija.

[18] Fazarinc R (2002). Solčava, Luče, Ljubno, Nazarje, Mozirje - Bodo kdaj varna pred naraso Savinjo?. Slov. vodar.

[19] ARSO. *Meterološki Portal*. http://meteo.arso.gov.si/met/sl/ (2019).

[20] ARSO. *Vodnatost rek v letu 2020*. https://www.arso.gov.si/vode/poročilainpublikacije/Vodnatostrekvletu2020.pdf (2020).

[21] Bat M, Uhan J, Bat M (2003). Tekoče vode. Vodno Bogastvo Slovenije.

[22] Kolbezen M, Pristov J (1998). Površinski Vodotoki in Vodna Bilanca Slovenije (Surface Streams and Water Balance of Slovenia).

[23] Buser, S. *Osnovna geološka karta SFRJ 1:100 000. Tolmač lista Celje: L 33–67*. (1979).

[24] Drovenik F, Pleničar M (1980). The origin of Slovenian ore deposits. Geologija.

[25] Salminen R (2005). Geochemical Atlas of Europe Part. 1, Background Information, Methodology and Maps.

[26] Kowalska JB, Mazurek R, Gąsiorek M, Zaleski T (2018). Pollution indices as useful tools for the comprehensive evaluation of the degree of soil contamination—a review. Environ. Geochem. Health.

[27] Tomlinson DC, Wilson DJ, Harris CR, Jeffrey DW (1980). Problem in heavy metals in Estuaries and the formation of pollution index. Helgol Wiss Meeresunlter.

[28] Wu W (2018). Assessment of heavy metal pollution and human health risks in urban soils around an electronics manufacturing facility. Sci. Total Environ..

[29] Müller G (1969). Index of geoaccumulation in sediments of the Rhine River. Geol. J..

[30] Förstner U, Ahlf W, Calmano W, Kersten M, Heling D (1990). Sediment criteria development. Sediments and Environmental Geochemistry.

[31] Ettler V (2006). Geochemical and Pb isotopic evidence for sources and dispersal of metal contamination in stream sediments from the mining and smelting district of Příbram, Czech Republic. Environ. Pollut..

[32] Dorotan D, Ozunu A, Costin D (2015). Accumulation of heavy metals in soils and alluvial deposits of Lăpuş river, Maramures county, Romania. Carpathian J. Earth Environ. Sci..

[33] Šajn R, Halamić J, Peh Z, Galović L, Alijagić J (2011). Assessment of the natural and anthropogenic sources of chemical elements in alluvial soils from the Drava River using multivariate statistical methods. J. Geochem. Explor..

[34] Hagemann L (2020). Potential hotspots of persistent organic pollutants in alluvial sediments of the meandering Wurm River, Germany. J. Soils Sedim..

[35] Vaněk A, Borůvka L, Drábek O, Mihaljevič M, Komárek M (2005). Mobility of lead, zinc and cadmium in alluvial soils heavily polluted by smelting industry. Plant Soil Environ..

[36] Izquierdo M, Tye AM, Chenery SR (2013). Lability, solubility and speciation of Cd, Pb and Zn in alluvial soils of the River Trent catchment UK. Environ. Sci. Process. Impacts.

[37] Adriano DC (1986). Trace Elements in the Terrestrial Environment.

[38] Kubier A, Wilkin RT, Pichler T (2019). Cadmium in soils and groundwater: A review. Appl. Geochem..

[39] Teran K, Žibret G, Fanetti M (2020). Impact of urbanization and steel mill emissions on elemental composition of street dust and corresponding particle characterization. J. Hazard. Mater..

[40] Stafilov T, Šajn R, Arapčeska M, Kungulovski I, Alijagić J (2020). Multi-element distribution in road and street dust in the Bitola region, North Macedonia. Geol. Maced..

[41] Pandey J, Singh R (2015). Heavy metals in sediments of Ganga River: Up- and downstream urban influences. Appl. Water Sci..

[42] Wojciechowska E, Nawrot N, Walkusz-Miotk J, Matej-Łukowicz K, Pazdro K (2019). Heavy metals in sediments of urban streams: Contamination and health risk assessment of influencing factors. Sustain.

[43] Atafar Z (2010). Effect of fertilizer application on soil heavy metal concentration. Environ. Monit. Assess..

[44] Šajn R (2005). Using attic dust and soil for the separation of anthropogenic and geogenic elemental distributions in an old metallurgic area (Celje, Slovenia). Geochem. Explor. Environ. Anal..

[45] Žibret G, Rokavec D (2010). Household dust and street sediment as an indicator of recent heavy metals in atmospheric emissions: A case study on a previously heavily contaminated area. Environ. Earth Sci..

[46] Huang L (2020). Heavy metals distribution, sources, and ecological risk assessment in Huixian Wetland, South China. Water (Switzerland).

[47] Balabanova B, Stafilov T, Šajn R, Tănăselia C (2016). Geochemical hunting of lithogenic and anthropogenic impacts on polymetallic distribution (Bregalnica river basin, Republic of Macedonia). J. Environ. Sci. Health Part A Toxic Hazard. Subst. Environ. Eng..

[48] Domitrovič-Uranjek D, Dobnik F (1990). Onesnaženost Okolja v Celju (Pollution of Environemnt in Celje).

[49] Pain DJ, Sánchez A, Meharg AA (1998). The Donana ecological disaster: Contamination of a world heritage estuarine marsh ecosystem with acidified pyrite mine waste. Sci. Total Environ..

[50] Vrhovnik P, Šmuc NR, Dolenec T, Serafimovski T, Dolenec M (2013). Impact of Pb-Zn mining activity on surficial sediments of Lake Kalimanci (FYR Macedonia). Turk. J. Earth Sci..

[51] Byrne P (2018). Water quality impacts and river system recovery following the 2014 Mount Polley mine tailings dam spill, British Columbia, Canada. Appl. Geochem..

[52] Duarte EB (2021). Trace metals in Rio Doce sediments before and after the collapse of the Fundão iron ore tailing dam, Southeastern Brazil. Chemosphere.

[53] Foucher D, Ogrinc N, Hintelmann H (2009). Tracing mercury contamination from the Idrija mining region (Slovenia) to the Gulf of Trieste using Hg isotope ratio measurements. Environ. Sci. Technol..

[54] Periáñez R (2013). Water circulation, sediment transport, and pollutant dynamics in Southern Iberia waters: A review on numerical modelling studies. ISRN Oceanogr..

[55] Saint-Laurent D, Lavoie L, Drouin A, St-Laurent J, Ghaleb B (2010). Floodplain sedimentation rates, soil properties and recent flood history in southern Québec. Glob. Planet. Change.

[56] URSZR (2019). Ocena Ogroženosti Zahodno Štajerske Zaradi Poplav.

[57] Grilc V, Grabne B, Ribarič Lasnik C (2013). Priprava onesnaženega zemljišča Stare Cinkarne v Celju na sanacijo (Preparatory works on remediation of contamianted site Old Zincworks in Celje). Onesnaženost Okolja in Naravni Vir Kot Omejitveni Dejavnik Razvoja v Sloveniji-Celjska Kotlina Kot Modelni Pristop za Degradirana Območja (Environmental Pollution and Natural Resources as a Limiting Favtor for Degraded Areas).

[58] Motel, P., Bronowska, K. & Szasz, L. *ENVIRON (PL 1091) - Phase II Environmental Site Assessment of the Cinkarna Metalurško-kemična industrija Sites in Celje and Mozirje*. https://civilne-iniciative-celja.si/dokumentacija/cinkarna-celje-vedda-2-final-report-20141203.pdf (2014).

[59] He D, Shi X, Wu D (2016). Particle-size distribution characteristics and pollution of heavy metals in the surface sediments of Kuitun River in Xinjiang, China. Environ. Earth Sci..

