# Supplementary material for: Distribution of Pb, Zn and Cd in stream and alluvial sediments in the area with past Zn smelting operations
Source: Sci Rep. 2021 Sep 2;11:17629. doi: 10.1038/s41598-021-96989-y (PMC8413453; doi:10.1038/s41598-021-96989-y)
Supplement: Supplementary file 1 — Supplementary Information 1. [file 41598_2021_96989_MOESM1_ESM.docx]

**Appendix 1.** Pb, Zn and Cd levels in stream and alluvial sediments in two fractions (less than 0.063 mm and between 0.063 and 0.125 mm) expressed in mg/kg.

|  | No. | Longitude | Latitude | Pb (< 0.063 mm)  (mg/kg) | Pb (0.063-0.125 mm)  (mg/kg) | Zn (< 0.063 mm)  (mg/kg) | Zn (0.063-0.125 mm)  (mg/kg) | Cd (< 0.063 mm)  (mg/kg) | Cd (0.063-0.125 mm)  (mg/kg) |
| --- | --- | --- | --- | --- | --- | --- | --- | --- | --- |
| Stream sediments | S01 | 14.6476 | 46.4195 | 21 | 14 | 69 | 60 | 0.14 | 0.12 |
|  | S02 | 14.7274 | 46.3796 | 16 | 12 | 76 | 56 | 0.31 | 0.20 |
|  | S03 | 14.7387 | 46.3595 | 18 | 12 | 68 | 60 | 0.18 | 0.12 |
|  | S04 | 14.7489 | 46.3552 | 13 | 10 | 64 | 64 | 0.21 | 0.23 |
|  | S05 | 14.8053 | 46.3622 | 13 | 12 | 58 | 48 | 0.16 | 0.13 |
|  | S06 | 14.8344 | 46.3397 | 16 | 11 | 54 | 55 | 0.17 | 0.21 |
|  | S07 | 14.8870 | 46.3105 | 14 | 15 | 63 | 57 | 0.18 | 0.18 |
|  | S08 | 14.9254 | 46.3129 | 12 | 11 | 54 | 53 | 0.11 | 0.19 |
|  | S09 | 14.9651 | 46.3369 | 25 | 27 | 83 | 82 | 0.39 | 0.43 |
|  | S10 | 15.0173 | 46.3172 | 18 | 13 | 59 | 53 | 0.21 | 0.16 |
|  | S11 | 15.0801 | 46.2648 | 19 | 26 | 77 | 79 | 0.30 | 0.29 |
|  | S12 | 15.1414 | 46.2386 | 30 | 37 | 105 | 115 | 0.35 | 0.73 |
|  | S13 | 15.2262 | 46.2333 | 33 | 34 | 111 | 121 | 0.49 | 0.61 |
|  | S14 | 15.2665 | 46.2244 | 33 | 32 | 120 | 109 | 0.47 | 0.51 |
|  | S15 | 15.2645 | 46.2188 | 48 | 49 | 270 | 257 | 1.81 | 1.56 |
|  | S16 | 15.2380 | 46.2017 | 41 | 52 | 214 | 274 | 1.99 | 2.27 |
|  | S17 | 15.2312 | 46.1922 | 38 | 41 | 164 | 195 | 1.19 | 1.40 |
|  | S18 | 15.2333 | 46.1643 | 32 | 34 | 159 | 169 | 1.18 | 1.48 |
|  | S19 | 15.2392 | 46.1465 | 34 | 35 | 163 | 172 | 1.13 | 1.26 |
|  | S20 | 15.1965 | 46.1334 | 34 | 19 | 111 | 105 | 0.58 | 0.44 |
|  | S21 | 15.2109 | 46.1214 | 36 | 36 | 156 | 166 | 1.16 | 1.23 |
|  | S22 | 15.2190 | 46.1089 | 30 | 32 | 139 | 135 | 0.74 | 0.70 |
|  | S23 | 15.1897 | 46.0893 | 30 | 29 | 117 | 85 | 0.72 | 0.52 |
|  | V01 | 15.2689 | 46.2262 | 89 | 96 | 522 | 402 | 3.72 | 2.80 |
|  | V02 | 15.2961 | 46.2298 | 35 | 24 | 155 | 103 | 0.69 | 0.36 |
|  | V03 | 15.3099 | 46.2236 | 39 | 31 | 188 | 149 | 0.65 | 0.52 |
|  | V04 | 14.6476 | 46.4195 | 22 | 13 | 81 | 63 | 0.28 | 0.22 |
|  | V05 | 14.7274 | 46.3796 | 19 | 16 | 92 | 68 | 0.32 | 0.29 |
|  | V06 | 14.7387 | 46.3595 | 17 | 12 | 64 | 42 | 0.28 | 0.19 |
|  | V07 | 14.7489 | 46.3552 | 15 | 13 | 48 | 39 | 0.17 | 0.12 |

|  | No. | Longitude | Latitude | Depth  (cm) | Pb (< 0.063 mm)  (mg/kg) | Pb (0.063-0.125 mm)  (mg/kg) | Zn (< 0.063 mm)  (mg/kg) | Zn (0.063-0.125 mm)  (mg/kg) | Cd (< 0.063 mm)  (mg/kg) | Cd (0.063-0.125 mm)  (mg/kg) |
| --- | --- | --- | --- | --- | --- | --- | --- | --- | --- | --- |
| Alluvial sediments | SP01 | 15.2157 | 46.2330 | 0-20 | 40 | 31 | 168 | 142 | 1.30 | 1.08 |
|  | SP01 | 15.2157 | 46.2330 | 20-40 | 37 | 34 | 165 | 153 | 1.78 | 1.45 |
|  | SP01 | 15.2157 | 46.2330 | 40-60 | 28 | 25 | 123 | 103 | 0.97 | 0.87 |
|  | SP01 | 15.2157 | 46.2330 | 60-80 | 27 | 23 | 103 | 90 | 0.74 | 0.65 |
|  | SP01 | 15.2157 | 46.2330 | 80-100 | 26 | 18 | 88 | 69 | 0.36 | 0.28 |
|  | SP02 | 15.2399 | 46.1971 | 0-20 | 29 | 22 | 99 | 100 | 0.37 | 0.45 |
|  | SP02 | 15.2399 | 46.1971 | 20-40 | 32 | 25 | 115 | 90 | 0.50 | 0.40 |
|  | SP02 | 15.2399 | 46.1971 | 40-60 | 28 | 21 | 100 | 79 | 0.38 | 0.24 |
|  | SP02 | 15.2399 | 46.1971 | 60-80 | 57 | 34 | 201 | 132 | 0.87 | 0.50 |
|  | SP02 | 15.2399 | 46.1971 | 80-100 | 68 | 49 | 221 | 177 | 1.11 | 0.83 |
|  | SP02 | 15.2399 | 46.1971 | 100-120 | 60 | 41 | 182 | 140 | 0.94 | 0.64 |
|  | SP02 | 15.2399 | 46.1971 | 120-140 | 66 | 52 | 204 | 149 | 1.03 | 0.82 |
|  | VP01 | 15.2709 | 46.2306 | 0-20 | 270 | 200 | 1690 | 1200 | 4.89 | 3.49 |
|  | VP01 | 15.2709 | 46.2306 | 20-40 | 178 | 119 | 1290 | 932 | 4.36 | 3.24 |
|  | VP01 | 15.2709 | 46.2306 | 40-60 | 1360 | 966 | 8910 | 6670 | 9.78 | 7.28 |
|  | VP01 | 15.2709 | 46.2306 | 60-80 | 4520 | 3410 | 26800 | 22300 | 31.28 | 22.35 |
|  | VP01 | 15.2709 | 46.2306 | 80-100 | 4030 | 3390 | 23200 | 20100 | 26.29 | 22.83 |
|  | VP01 | 15.2709 | 46.2306 | 100-120 | 3760 | 2940 | 23100 | 20400 | 26.33 | 23.07 |
|  | VP01 | 15.2709 | 46.2306 | 120-140 | 4090 | 3330 | 23300 | 20500 | 21.52 | 17.88 |
|  | VP02 | 15.3523 | 46.2168 | 0-20 | 25 | 18 | 98 | 65 | 0.50 | 0.39 |
|  | VP02 | 15.3523 | 46.2168 | 20-40 | 29 | 19 | 117 | 75 | 0.73 | 0.42 |
|  | VP02 | 15.3523 | 46.2168 | 40-60 | 30 | 21 | 123 | 81 | 0.74 | 0.52 |
|  | VP02 | 15.3523 | 46.2168 | 60-80 | 30 | 21 | 119 | 77 | 0.87 | 0.49 |
